# Effect of Debinding Process on Aluminum Nitride Ceramics Fabrication via Digital Light Processing 3D Printing

**DOI:** 10.3390/polym17202769

**Published:** 2025-10-16

**Authors:** Ning Kuang, Wenjie Zhao, Junfei Wu

**Affiliations:** 1 College of Electromechanical Engineering, Qingdao University of Science and Technology, Qingdao 266061, China; kuang.ning@mails.qust.edu.cn; 2 College of Sino-German Science and Technology, Qingdao University of Science and Technology, Qingdao 266061, China

**Keywords:** 3D printing, additive manufacturing, DLP, aluminum nitride, debinding

## Abstract

Aluminum nitride (AlN) ceramics exhibit exceptional properties, making them attractive for a wide range of applications. To address the growing need for customized and geometrically intricate AlN components, digital light processing (DLP) has garnered significant interest as a highly promising additive manufacturing technology. In this study, the effects of the sintering process on DLP 3D printing of AlN ceramics were investigated. An optimized slurry formulation with a solid loading of 52 vol% was developed, exhibiting excellent rheological properties. By applying a controlled sintering heating rate of 0.5 °C/min, dense AlN ceramic components were successfully fabricated, achieving a bending strength of 212.6 MPa. This provides a novel approach for optimizing the DLP additive manufacturing process of AlN ceramics.

## 1. Introduction

Aluminum nitride (AlN) ceramic possesses a variety of excellent properties, including high thermal conductivity, excellent electrical insulation, high mechanical strength, and a low coefficient of thermal expansion, which renders it highly suitable for microelectronics, heat dissipation and transfer applications [1,2,3]. Notwithstanding its industrial importance, the manufacture of AlN components featuring complex geometries is impeded by conventional processing techniques. Methods such as dry pressing, injection molding, and gel casting are inherently limited by their dependence on rigid tooling [4,5,6,7,8], which limits their ability to produce parts with intricate internal features or freeform architectures.

As a type of additive manufacturing, also known as 3D printing technology, DLP (Digital Light Processing) has attracted growing interest. Given that layer-by-layer fabrication approach, this technology enables direct transformation of digital models into physical objects [9], overcoming the inherent limitations of traditional ceramic manufacturing processes. It offers unprecedented design flexibility for producing components with complex geometries, while also featuring rapid production cycles, high forming accuracy, and a significant reduction in material waste [10,11].

Nevertheless, the efficacy of the DLP process is critically contingent upon the rheological and photopolymerization characteristics of the AlN slurry [12,13]. Consequently, significant research efforts have been directed towards optimizing these properties to enhance both printability and the performance of the sintered ceramics. Xu et al. [14] investigated the influence of photosensitive resin composition and photoinitiator on the curing depth and width. Additionally, it was found that the addition of 3 wt% KH560 reduced the sedimentation rate and viscosity. Ultimately, an aluminum nitride slurry with a solid loading of 50% was prepared, and an AlN ceramic body with a bending strength of 107.2 MPa was obtained. Further studies by Lin et al. [15] employed a novel surfactant improved the dispersibility of aluminum nitride powder in the resin, resulting in a low-viscosity slurry with a solid loading exceeding 60 vol%, and ultimately yielding AlN ceramics with a dense and homogeneous microstructure. Subsequent work by Lin et al. [16] investigated the effect of yttrium nitrate content on the particle size of AlN powder, slurry viscosity, curing behavior of the suspension, as well as the properties and microstructure of sintered AlN ceramics, the results showed that it significantly reduced the viscosity of the suspension and increased the curing depth. Wang et al. [17] explored the effects of three different refractive index resins on the viscosity and curing behavior of AlN ceramic suspensions, a high-performance AlN ceramic with a relative density greater than 98% was successfully obtained. Additionally, Tang et al. [18] investigated the optimal dispersant content in AlN ceramic slurries and examined its effects on the shrinkage, density, and microstructural evolution of the resulting ceramics. Qi et al. [19] studied the effect of nitrate content on the viscosity and curing properties of AlN suspensions, thereby optimizing the curing process and successfully producing dense AlN ceramics. Xu et al. [20] optimized the particle size distribution, rheology, and curing kinetics of AlN slurries by systematically varying ball milling time. This approach produced a high solid loading slurry, which after debinding and sintering, achieved a high bending strength of 98.7 MPa in the resulting AlN ceramics.

As printed AlN ceramic green body requires thermal treatment, consisting of debinding and sintering. The debinding stage eliminates the cured photopolymerization resin, while sintering promotes densification through particle coalescence [21,22]. Therefore, the removal of organic binders from AlN green bodies is a critical step prior before sintering. This debinding process significantly influences the performance and microstructure of the final sintered ceramic components [23]. Alves et al. [24] examined the influence of debinding atmosphere (air or N_2_) for alumina ceramics additive manufacturing, it was found that the specimens that were debinding in nitrogen could achieve a higher density. Bezek et al. [25] conducted research on photopolymerization additive manufacturing of silica-based ceramics and, by balancing the debinding temperature and time, found an effective method to reduce the total debinding time. Meng et al. [26] investigated debinding process of zirconia ceramic via vat polymerization additive manufacturing, by taking advantage of the unique features of vacuum debinding and rapid heating with porous graphite felt to accelerate the removal of the binder, the complete debinding process was achieved within 30 min.

However, research on AlN ceramics has predominantly focused on slurry preparation, with significantly less attention paid to the thermal treatment of printed green bodies, particularly the debinding process.

Based on previous research on photosensitive resins [27], in this study, we further investigated the effect of debinding on the DLP 3D printing of AlN ceramics. Additionally, we also determined the optimal type and concentration of the dispersant. Furthermore, the solid loading of the AlN slurry was optimized to establish the suitable level for printing process. Finally, the optimized slurry formulation and debinding parameters were employed, resulting in the successful fabrication of AlN ceramic components with high bending strength.

## 2. Materials and Methods

### 2.1. Materials

In this study, aluminum nitride powder (D50 = 1 µm, Shanghai ST-Nano Science & Technology Co., Ltd., Shanghai, China) was used. Yttria powder (D50 = 1 µm, Shanghai ST-Nano Science & Technology Co., Ltd., Shanghai, China) was served as a sintering additive. The powders were modified using a surfactant 3-(trimethoxysilyl) propyl methacrylate (KH570, Jiangxi Chenguang New Materials Co., Ltd., Jiujiang, China). The photosensitive resin was composed of a mixture of monomers, including acryloyl morpholine (ACMO, Shanghai Guangyi Chemical Co., Ltd., Shanghai, China), diethylene glycol diacrylate (DEGDA, Guangzhou Goliang Technology Co., Ltd., Guangzhou, China), and trimethylolpropane triacrylate (TMPTA, Shanghai Guangyi Chemical Co., Ltd., Shanghai, China). The photoinitiator used was 2,4,6-trimethyl benzoyl diphenyl phosphine oxide (TPO, Shanghai Guangyi Chemical Co., Ltd., Shanghai, China). BYK111, BYK190 (both from BYK-Chemie GmbH, Wesel, Germany) and KOS110 (Dongguan Haoyouduo New Materials Co., Ltd., Dongguan, China) were employed as dispersants.

### 2.2. Powder Modification

**Figure 1:**
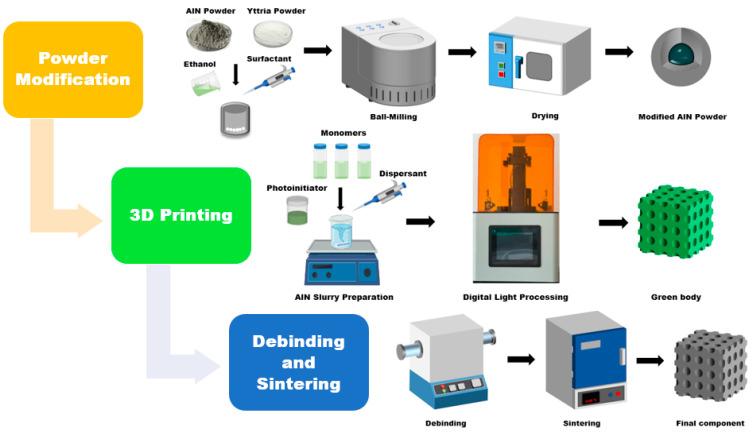
Three-dimensional printing AlN ceramic fabrication process.

The powder modification process is illustrated in Figure 1. A mixture of AlN and yttria powders with a mass ratio of 95:5 was first dispersed in ethanol. Then, 3 wt% of KH570 (relative to the total powder mass) was added as a surfactant. The suspension was subsequently processed by planetary ball milling (BQM-1L, Changsha Miqi Instruments & Equipment Co., Ltd., Changsha, China) at 300 rpm for 8 h to effectively break down agglomerates. After milling, the ethanol was removed by drying the suspension at 60 °C. The dried powder was finally sieved through a 100-mesh screen to ensure a uniform particle size distribution.

### 2.3. Preparation of AlN Ceramic Slurry

The photosensitive resin was mixed from three monomers, ACMO, DEGDA and TMPTA with mass ratio of 56.7%, 2.7% and 40.6%, respectively, TOP was added with 3 wt% of resins as the photoinitiator. The resin was mixed via a magnetic stirrer (RCT-Basic, IKA-Werke GmbH & Co. KG, Staufen im Breisgau, Germany) at 500 r/min for 60 min. After that, a predetermined amount of the modified powder was incorporated into the mixed photosensitive resin, dispersants BYK111, BYK190 and KOS110 with 1 wt% to 4 wt% of powder was added to the suspension, and the mixture was homogenized by ball milling for 10 min to obtain a uniform slurry.

### 2.4. Fabrication

AlN ceramic green bodies were fabricated using a DLP ceramic printer (BLD-25-C1, Qingdao Breuck 3D Additive Manufacturing Co., Ltd., Qingdao, China). The printing layer thickness was set to 30 µm, with an exposure power ranging from 5 to 9 mW/cm^2^ and an exposure time between 1 and 40 s. After printing, the green bodies underwent debinding in a vacuum tube furnace (OTF-1200X, Hefei Kejing Materials Technology Co., Ltd., Hefei, China) at heating rates varying from 0.5 to 5 °C/min up to 550 °C, followed by a 3 h dwell at that temperature. Subsequently, the samples were sintered in a nitrogen atmosphere furnace (model ZT-18-22, Shanghai Chenhua Technology Co., Ltd., Shanghai, China) at a heating rate of 5 °C/min up to 1800 °C and held for 4 h. The final AlN ceramic samples were obtained after cooling.

### 2.5. Characterization

The viscosity of the AlN slurries was measured using a rotary viscometer (NDJ-8Spro, Shanghai Xiniulab Instruments Co., Ltd., Shanghai, China). The three-point bending strength of the sintered ceramic specimens was evaluated on a digital electronic universal testing machine (WH-70, Ningbo Weiheng Testing Instruments Co., Ltd., Ningbo, China) at a loading rate of 0.5 mm/min. The microstructure of the sintered specimens was characterized by scanning electron microscopy (SEM, Regulus8100, Hitachi, Tokyo, Japan). The Vickers hardness was measured by Vickers hardness tester (THVP-50, Kejing Auto-Instrument Co., Ltd., Shenyang, China) with loading force of 50 kg and loading time of 10 s.

## 3. Results and Discussion

### 3.1. Rheological Properties of Photopolymer Resin

**Figure 2:**
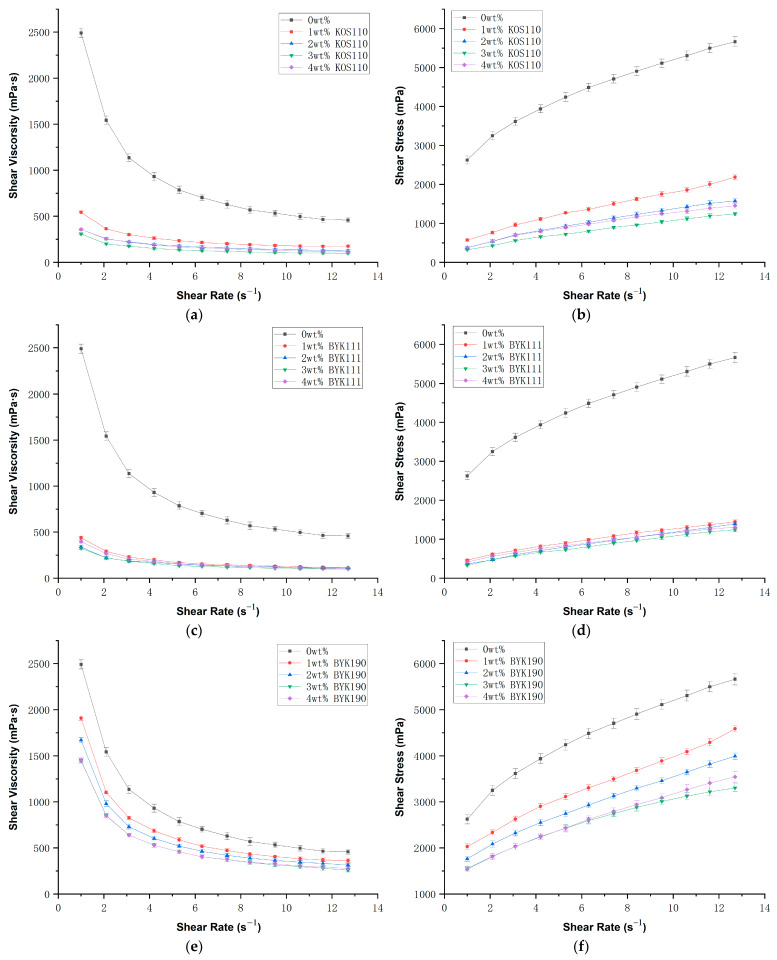
Relationship of shear viscosity, shear stress and shear rate with different dispersant content using a 20 vol% AlN ceramic slurry. (**a**,**b**) KOS110; (**c**,**d**) BYK111; (**e**,**f**) BYK190.

The selection of an appropriate dispersant and its optimal concentration is critical for attaining the desired rheological properties in high solid loading slurries used in ceramic additive manufacturing. To formulate an aluminum nitride ceramic slurry with low viscosity and favorable dispersion characteristics, three distinct dispersants KOS110, BYK111 and BYK190 were tested, respectively, in this study. Meanwhile, to determine the optimal dispersant concentration for the aluminum nitride slurry, we investigated the rheological properties of suspensions with different dispersant concentrations (specifically 0 wt%, 1 wt%, 2 wt%, 3 wt%, and 4 wt% relative to the modified AlN powder), as shown in Figure 2 For comparative purposes, a slurry with a solid loading of 20 vol% was used.

As illustrated in Figure 2a, regardless of the concentration of the dispersant, the viscosity of the slurry containing dispersant KOS110 is significantly lower than that of the slurry without the dispersant, and the viscosity decreases with increasing shear rate, indicating non-Newtonian fluid behavior. As the dispersant content rises from 1 wt% to 3 wt%, the viscosity decreases. However, a further increase to 4 wt% leads to a rise in viscosity. As shown in Figure 2b, shear stress decreases as the dispersant content increases from 1 wt% to 3 wt%, but rises again when the content reaches 4 wt%. This phenomenon is commonly attributed to molecular flocculation caused by excessive dispersant addition, which reduces the fluidity of the suspension [28,29].

As shown in Figure 2c,d, the slurry containing dispersant BYK111 demonstrated behavior similar to that with KOS110, achieving minimum viscosity and shear stress at a concentration of 3 wt% BYK111. In contrast to KOS110, however, the influence of varying dispersant content was less significant.

Figure 2e,f present the rheological behavior of the slurry prepared with dispersant BYK190. In comparison to KOS110 and BYK111, dispersant BYK190 exhibits a weaker effect on viscosity reduction. Nevertheless, a dispersant content of 3 wt% still achieves the optimal dispersion performance.

**Figure 3:**
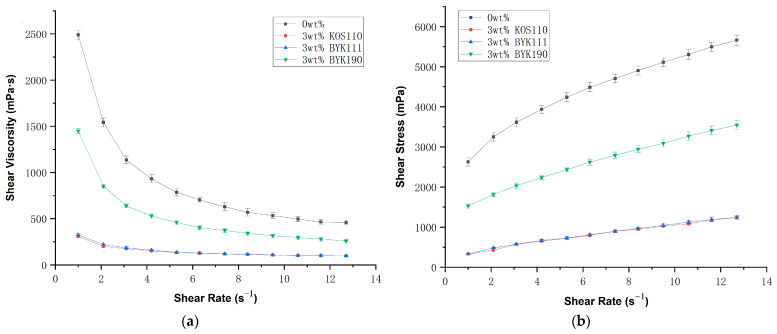
(**a**) Relationship between shear viscosity and shear rate of AlN ceramic slurry with different dispersant; (**b**) Relationship between shear stress and shear rate of AlN ceramic slurry with different dispersant.

The optimal concentrations of the three dispersants were selected for comparison. As illustrated in Figure 3, the slurries prepared with dispersants KOS110 and BYK111 exhibited the lowest viscosity and demonstrated superior rheological properties. Although both KOS110 and BYK111 at 3 wt% provided nearly identical improvements in rheological performance, BYK111 showed an overall better enhancement effect, as evidenced in Figure 2c. Therefore, 3 wt% BYK111 was chosen for subsequent studies.

**Figure 4:**
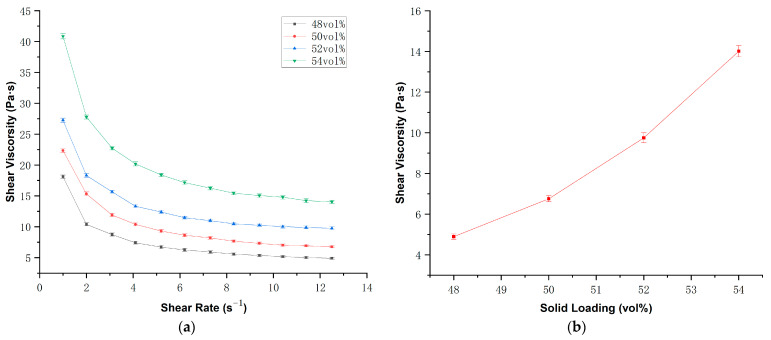
(**a**) Relationship between shear viscosity and shear rate of AlN ceramic slurry with different solid loading; (**b**) shear viscosities of the AlN ceramic slurry with different solid loading at a shear rate of 12 s^−1^.

Under identical photosensitive resin formulation and dispersant content conditions, the solid loading of ceramic powder fundamentally governs the rheological properties of the slurry. A systematic characterization of the AlN ceramic suspensions was conducted, identifying an optimal range of solid loading, as illustrated in Figure 4a. It was observed that viscosity increases markedly with solid loading, with a particularly sharp increase occurring at 54 vol%. Figure 4b present the viscosities with different solid loading at same shear rate of 12 s^−1^. Based on our previous experience, the viscosity should not exceed 10 Pa·s at a shear rate of 12 s^−1^, to meet the requirements for the fluidity of printing. However, the slurry with 54 vol% solid loading exhibited a viscosity of 14 Pa·s under the same shear rate condition, surpassing the printable threshold. Therefore, a 52 vol% solid loading slurry was selected for the subsequent tests.

### 3.2. Photocuring Performance of AlN Ceramic Slurry

The photocuring process of the AlN ceramic slurry begins when the photoinitiator in the photosensitive resin system generates free radicals upon light exposure, initiating monomer polymerization. This results in the polymerization and crosslinking of reactive monomers, forming a three-dimensional network that encapsulates the ceramic powder.

The cure depth C_d_ (μm) of the slurry depends on the energy dose and can be modeled by the Beer–Lambert law [30], which describes light attenuation in terms of material properties. Based on this principle, the energy transmitted through the slurry decreases with depth, leading to a reduction in cure depth as the layer thickness increases. The relationship between cure depth and energy dose is given by the following expression:

(1) $$C_{d}=D_{p}\ln\left(\frac{E}{E_{c}}\right)$$

In this equation, the projection depth D_p_ (μm) denotes the penetration depth of the optical system, E_c_ (mJ·cm^−2^) refers to the critical energy required for curing, and E (mJ·cm^−2^) represents the actual exposure energy applied. The expression shows that cure depth increases logarithmically with energy dose.

**Figure 5:**
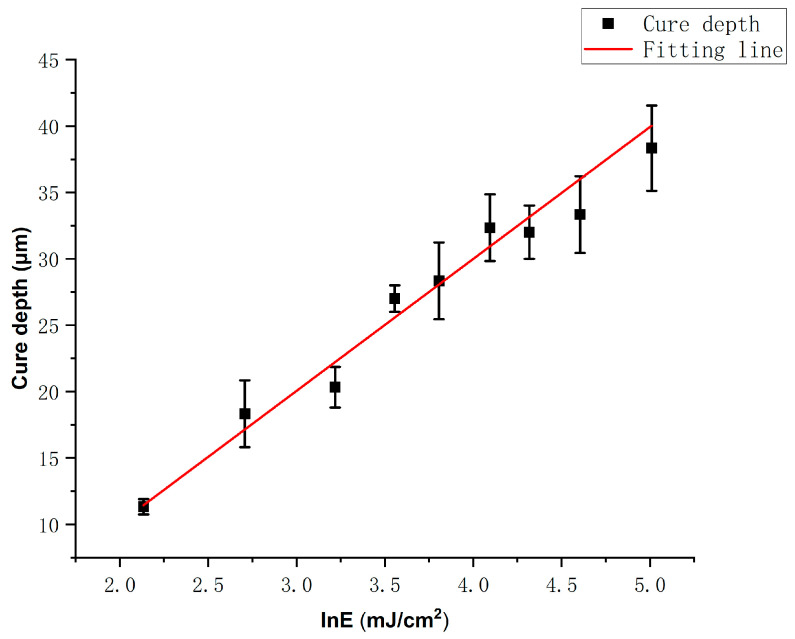
Linear fitting relationship between exposure energy and cure depth of the AlN slurry.

In this study, the relationship between C_d_ and lnE was analyzed, as presented in Figure 5. The experimental cure depths exceeded the 30 μm layer thickness when the exposure energy E_c_ was greater than 60 mJ·cm^−2^, thereby meeting the essential printing requirements. When the exposure energy reached 150 mJ·cm^−2^, the cure depth reached 40 μm. The increase in cure thickness was not substantial, which can be attributed to significant scattering and absorption of light by the AlN powder as the cure depth increases. This leads to pronounced attenuation of incident light radiation within the slurry [31]. Furthermore, curve fitting was performed, yielding a corresponding R^2^ value of 0.98385, which demonstrates the high accuracy of the model.

### 3.3. Debinding

**Figure 6:**
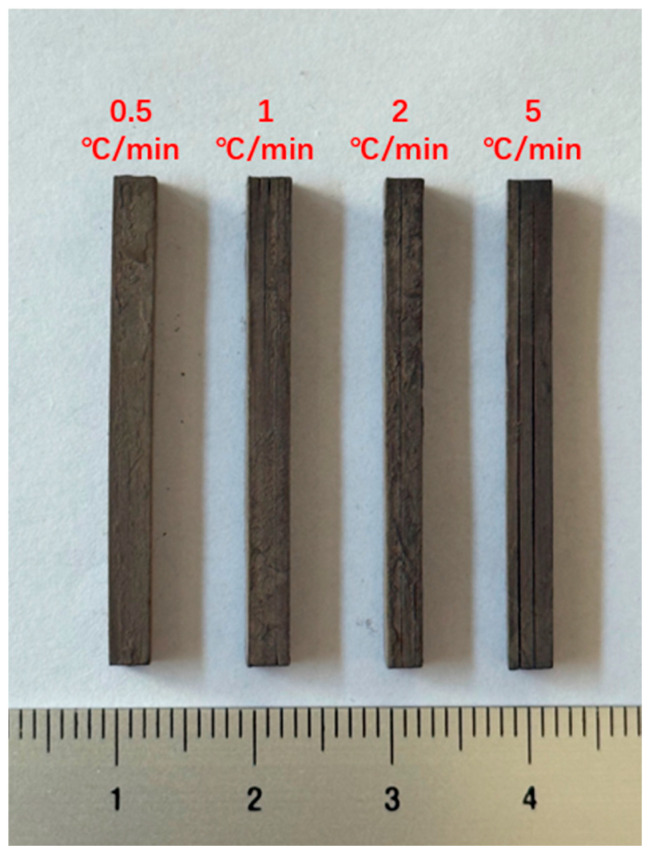
Debound AlN specimens with different heating rates.

Based on the thermogravimetric analysis curve obtained from a previous study [27], the same printed specimens were subjected to debinding tests under the conditions of a maximum temperature of 550 °C and a holding time of 3 h, using heating rates of 0.5 °C/min, 1 °C/min, 2 °C/min and 5 °C/min, respectively. As shown in Figure 6, the specimen with a heating rate of 0.5 °C/min remained relatively intact with no obvious cracks observed. Specimens heated at rates of 1 °C/min and 2 °C/min exhibited minor cracks, while the specimen with a heating rate of 5 °C/min showed multiple clearly visible cracks.

This is because the gases generated during resin decomposition typically diffuse through the fine pores between ceramic particles [32]. When the rate of gas generation exceeds the rate of diffusion, the trapped gases lead to a continuous increase in internal pressure. Excessively high heating rates cause the rapid vaporization and decomposition of organic components, resulting in extremely high internal pressure that can induce critical defects such as blistering, cracking, and delamination in the parts [33,34,35,36]. Therefore, employing a lower heating rate effectively mitigates crack initiation during debinding. While this approach increases processing time, the overall impact remains minimal due to the relatively low peak temperature required for debinding. As a result, the debinding process employing a heating rate of 0.5 °C/min was adopted in this study for subsequent sintering and further testing.

### 3.4. Properties of AlN Ceramics

**Figure 7:**
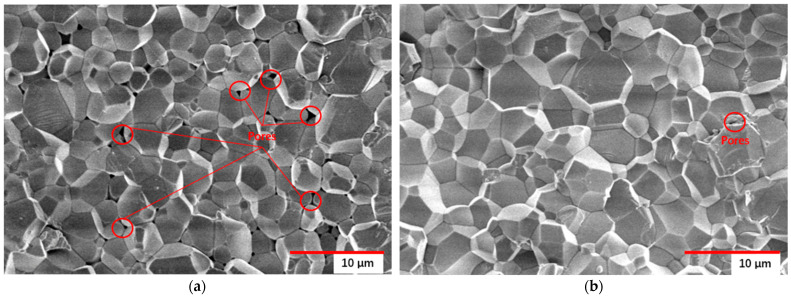
Microstructure of sintered AlN ceramic. (**a**) 50 vol% solid loading; (**b**) 52 vol% solid loading.

The mechanical properties of the sintered AlN ceramics were assessed using three-point bending tests, 5 specimens were tested, yielding a bending strength of 212.60 ± 69.17 MPa. The Vickers hardness of the specimen was 1010.5 ± 157.5 HV, as measured at three points. As shown in the SEM micrograph in Figure 7, compared to the microscopic image (Figure 7a) of 50 vol% solid loading in our previous study [27], which shows a significant number of pores, the microscopic image (Figure 7b) of 52 vol% solid loading exhibits a noticeable reduction in porosity, the fracture surface of the specimen exhibits a nearly pore-free, highly densified microstructure, which corroborates the observed high bending strength, demonstrating that an increase in solid content contributes to enhancing the density of AlN ceramics.

These results further demonstrate that the optimized resin formulation and debinding process developed in this study are well-suited for the DLP additive manufacturing of AlN ceramics, contributing to reduced defect formation and improved mechanical performance.

## 4. Conclusions

In this study, the effect of the heating rate during the debinding process was investigated, and the causes of related defects were analyzed. The influences of dispersant type, content, and solid loading on the rheological properties of the AlN ceramic slurry were also examined.

1. Dispersant impact: this study clarified the crucial function of dispersants in regulating rheological properties, with the formulation containing 3 wt% BYK111 exhibiting the lowest viscosity across all evaluated compositions.
2. Solid loading impact: a slurry with enhanced rheological properties was prepared by adding 3 wt% BYK111, achieving a high solid loading of 52 vol%.
3. Debinding process impact: this study demonstrates that employing a slower heating rate during debinding facilitates the removal of gases from the green body, thereby mitigating crack formation. Using green bodies printed with the optimized slurry and subjected to a debinding process at a heating rate of 0.5 °C/min, followed by sintering, dense aluminum nitride ceramic components were successfully obtained, achieving a bending strength of 212.6 MPa, and Vickers hardness of 1010.5 HV.

## Data Availability

The original contributions presented in this study are included in the article. Further inquiries can be directed to the corresponding authors.
